# Cases of Pulmonic Disease Treated by a Regulated Temperature

**Published:** 1813-05

**Authors:** Isaac Buxton

**Affiliations:** Physician to the London Hospital


*For the Medical and Physical Journal.*

### CASES *of* PULMONIC DISEASE *treated by a* REGULATED TEMPERATURE.

*By* ISAAC BUXTON, M.D. *Physician to the London Hospital.*

DURING the winter months I have, in many instances of disease in the chest, endeavored to persuade patients to heat the rooms which they inhabited to about 65°, but, in general, the recommendation was not at all attended to, or was carried on in a manner so very imperfect, as scarcely to afford any relief to the patient. In the cases of several patients, an account of whom was inserted in the Ed. Med. Journal for October 1811, the circumstances just mentioned are observable; and in many other instances the same have occurred. A few individuals, however, it should be remarked, have attended strictly to the recommendation, and generally experienced results extremely satisfactory. Wishing to have more examples of this kind at the same time, and immediately under my own management, I procured a room at No. 5, Freeman's-lane, Horselydown, and there placed several patients, whose cases I shall presently relate. This room was 15 feet by 14½, and was heated by what, in this country, is called a German stove, which projected into the room 5½ feet. A great part of the time during which I occupied this room, a thermometer was hung up about 9½ feet from the stove. The patients were directed to keep this instrument as nearly as possible at 65°. I went into the room at almost all hours of the day, and seldom failed to note on paper the height of the thermometer. It generally stood very nearly at the degree desired. I never but once saw it so low as 61°; and on one occasion it was as high as 71°. On removing the thermometer to the chimney-piece, it usually rose in 10 minutes to 70° or 72°. When the patients went to bed at night the fire was made up, and was permitted to burn out. From these observations, I suppose that the temperature of the air which they breathed was generally between 60° and 70°.

Several medical gentlemen did me the favor to visit this apartment. Their names I need not mention. I should, however, be deficient in gratitude, were I to omit returning my sincere acknowledgments to Mr. Key, of Fair-street, Horselydown, for the very liberal and kind assistance he gave me in attending the patients.

I shall now relate, in the order of time, the cases of those who were admitted into this room.

CASE

Case I.—John Osborn, hatter, aged 21½, residing at No. 3, Maze Pond, Southwark; ill six weeks; admitted Jan. 2d.—He is extremely hoarse; coughs much both by day and night; expectoration is considerable; has no particular pain in any part of the chest; is worse when the weather is cold or foggy; cough and other symptoms aggravated if he goes into a cold place after having been in one which is warmer; is very weak; continues to work at his business, but with considerable difficulty. He attributes his disorder to a cold which he caught after having had his hair cut. On the evening of the same day, while perspiring, he was exposed to rain. Since that time his complaints have been nearly the same as those above described, but they have varied in degree. At the commencement of his disorder he had a pain under his left shoulder-blade; but this has long quitted him.

Liq. Ant. tart. ter die.

6th.—Rather better; but hoarseness and cough continue.

Empl. Lytt. Scrob. Cord. et Cerat. Sabin.
Liq. Ant. tart. Tr. Scill.

8th.—Still better; yet the action of the blister does not appear to have produced any material difference. Blistered part quite well. Pr.

10th.—On the 8th, the weather being extremely mild, I permitted him to take a walk. When he quitted the chamber he coughed as he had been accustomed to do, before his admission, on exposure to the air. While he was without doors, although but for a short time, his cough was as troublesome as it has sometimes been during a whole day when he continued in the heated room. It rained a little, as he was walking, and he was wetted in a trifling degree. His cough, dyspnœa, and hoarseness, were much aggravated yesterday; they still continue aggravated, though not to so great an extent.

Liq. Ant. tartar.
Empl. Lytt. Scrob. Cord. et Cer. Sabin.

12th.—Better in every respect; blister continues to draw.

Pr. Cer. Sabin.

15th.—Blister still open; hoarseness much diminished. Yesterday felt himself remarkably well; scarcely coughed throughout the whole day; but this day coughs rather more. Pr.

17th.—Rather better; blister scarcely draws. Pr.

Unguent. Cetacei.

19th.—Nearly as he was.

Liq. Ant. tart. c. Tr. Scill.
Empl. Lytt. Stern. et Cer. Sabin.

22d.—The day before yesterday in the morning his hoarseness and cough were much increased; for this he could assign no cause; was as well as usual the night before. The blister was not put on till the increase of disease had commenced. Pulse now rather quick; hoarseness greater than when I saw him last, but not so severe as yesterday and the day before; did not cough much this morning; blister does not discharge much. Pr.

24th.—Hoarseness still more diminished; on the whole he is rather better; blister discharges but little. Pr.

27th.—The night before last the cough troubled him much, and he expectorated more than usual. In the evening of yesterday was better, and still continues so. Expectoration much diminished; blister scarcely discharges at all, but the part is very red. Pr. Ung. Cetacei.

29th.—Nearly as he was. Pr.

30th.—Osborn says, that his cough is now very much better than when he was admitted, particularly during the day; expectoration and breath also considerably relieved; hoarseness relieved, but not to so great a degree as either of the other symptoms. He is not so well as he was about twelve days ago; but is not at all worse than he has been for some days past. Pr. Discharged.

Case II.—James Ellis, laborer, aged 55; ill five months; admitted Jan. 13th.—Coughs very much, particularly during the night; breath always extremely short, but occasionally for an hour or two much worse than at other times; expectoration considerable, being about a pint in 24 hours; it is colored red; is frequently brought up with great difficulty; he has pain in his left side; also, in an inferior degree, in his chest and back; occasional stitches in his chest; cannot lie down, excepting on his right side; is not hoarse; head aches occasionally; appetite not bad; rather thirsty; bowels regular; is excessively weak; tongue clean; pulse small, not quick. He feels best in the morning. During the night is almost totally deprived of sleep by violent fits of coughing, which continue for one or two hours, and recur at very short intervals. The disorder is aggravated in cold or foggy weather, or when he quits a warm for a cold situation. The severity of his disorder, joined to great debility, renders him totally unable to work. Previous to April of last year he was perfectly well. In that month he caught a severe cold, and was ill two or three months; was then better during five or six weeks; then again in August caught cold, and became extremely ill, and since that period the disorder has progressively increased. About three months ago he spat blood.

Liq. Ant. tart.

Empl. Lytt. Hypoch. sin. et Cerat. Sabin.

15th.

15th.—The blister was not put on till yesterday; it has risen well, and discharges. Ellis expectorates much mucus mixed with blood; breath, cough, and expectoration, better. Pr.

17th.—Does not spit more than half a pint in 24 hours; expectoration of an uniform pink color. Pr.

19th.—Blister discharges; expectoration more diminished; color the same; some parts redder than the rest; is still waked occasionally in the night by the cough, but the fit of coughing is over in five or ten minutes, and then he sleeps again. Pr.

22d.—Breath, cough, and expectoration, better; bowels regular; appetite moderate; is scarcely awakened by the cough; blister discharges. Pr.

24th.—Blister discharges; expectoration farther diminished in quantity, but its color a deeper red; coughs still less; breath nearly as last report; still feels occasional stitches. Pr.

27th.—Cough almost gone; it scarcely ever disturbs him; breath nearly as it was; blistered surface almost well; expectoration still pink; medicine this morning made him sick, an effect it never before produced; some head-ach during two or three days; feels occasional pain between his shoulders; pulse moderate. Pr.

31st.—Blister quite well; still feels pains in his chest, side, and between the shoulders, when he coughs; expectoration more like simple mucus, being less opaque, and less colored; quantity smaller; pulse soft and small, not particularly quick; bowels regular. Pr.

Empl. Lytt. stern. et Cer. Sabin.

Feb. 3d.—Better; had severe pain in the head last night and this morning, but that is gone off; bowels rather confined; pains about chest and shoulders diminished; does not expectorate more than half a wine-glass in two nights; expectoration scarcely at all colored; blister discharges; breath generally well, but occasionally very short. Pr.

Pil. Al. c. Myrrh. j. alt. noct.

5th.—Much more coughing and expectoration this morning than usual; pain in chest better, but still occasionally feels it, chiefly in the left side; fits of dyspnœa do not attack him so often as they did; they continue about an hour, a period of time less than formerly; blistered surface nearly well. Pr. Empl. Lytt. Hyp. sin. et Cerat. Sabin.

7th.—Expectoration trifling; none during the night; blister discharges; scarcely coughed at all since the day before yesterday, excepting this morning, when he coughed much for about half an hour; has occasional shifting pains in his chest; no fixed pain in the left side, and scarcely any in the shoulders; shoulders; fits of dypsnœa diminish, they recur perhaps three times a-day for about half an hour; they attack him chiefly after he has taken victuals; scarcely coughs at that time, if he does, has no expectoration; tongue clean. Pr.

14th.—Blistered surface not quite well; occasional pains between the shoulders; chest pains him a little; fits of dyspnœa just as a week ago; appetite good; expectoration deep-colored, but not red, and trifling in quantity. Pr.

16th.—Pain in left side of the head; small of back very weak; cough still further diminished; breath nearly as last report; bowels rather confined till he took a pill last night. Pr.

Empl. Pic. Comp. Lumb.

19th.—Was not within when I visited the patients. Pr.

20th.—Discharged.

Case III.—Richard Hughes, cooper, aged 20, residing at No. 40, Salisbury-lane, Bermondsey; ill eight months; admitted Jan. 20th.—Coughs much; breath very short; raises much mucus, which he expectorates easily; perspires both in the day and night; is very weak; tongue clean; bowels regular. He has been under my care since Sept. 12th, 1812; at that time his symptoms were nearly the same as they now are, excepting that they were then worse. Whilst I attended him previous to his admission, he had a succession of perpetual blisters, Liq. Ant. tart. et Tr. Scill. with Pil. Colocynth. his bowels having been confined occasionally. Under this treatment the symptoms, excepting the dyspnœa, gradually amended till about the middle of December, when his complaints were aggravated. At the time of his admission, he had returned to nearly the same state in which he was previous to this increase of disease.

Rep. Liq. Ant. tart. c. Tr. Scill. et Cerat. Sabin. part. vesicat.

22d.—Better; cough not so bad; expectoration diminished; tongue clean; perspires both day and night more than before his admission; hands at this moment quite moist; bowels open once or twice daily; pulse quick; appetite good. Pr.

24th.—Breath no better; coughs less; perspiration not so great; blister discharges. Pr.

27th.—Breath improved; appetite good, just as before his admission; scarcely coughs at all; perspires, but less than he did; weaker than when admitted; tongue clean; blister quite well. Acid. Sulphur.

31st.—Breath as before; cough of no consequence; does not perspire so much, chiefly in his hands; stronger; tongue clean; bowels regular; a little pain occasionally in the left side of his chest; pulse regular, but rather quick. Pr.

Feb.

Feb. 3d.—Breath somewhat improved; cough has totally left him, except perhaps once in the morning, does not then expectorate; still pain occasionally in the left side; perspiration not quite so great; is rather stronger. Pr.

Empl. Lytt. Hyp. sin. et Cerat. Sabin.

5th.—Breath rather better; pain in side diminished; blister draws. Pr.

7th.—Breath extremely short last night, more so than it has been a considerable time past; the dyspnœa commenced about nine, and lasted perhaps an hour; the remainder of the night not particularly affected; did not cough; perspiration as four days ago; rather stronger; appetite good; blister discharges. Pr.

14th.—Breath better; has some wheezing in his throat; no cough; rather stronger; perspiration diminished; blister well. Pr.

16th.—Wheezing nearly as before; breath still better; strength further increased; does not perspire so much. Pr.

17th.—Discharged.

Case IV.—John Corney, news carrier, aged 17, residing at No. 5, Three-crown-court, Whitechapel; ill six months; admitted Jan. 20th.—Appears younger than he really is; is delicate, and narrow-chested. He coughs much; at that time feels pain about his throat; wheezes; vomits occasionally from the violence of the cough; what he brings up is sometimes bitter; does not vomit from any other cause; expectorates about a wine-glass full in 24 hours; is excessively weak; has wasted away considerably; pulse quick. I have attended him occasionally since Sept. 23d, 1812. At that period his cough and breath were worse than they now are, but he did not appear so thin and weak as he is at present. The remedies have been laxatives, to prevent the bowels from being confined, perpetual blisters, antimony, squills, and sulphuric acid.

Rep. Acid. Sulphur.

Empl. Lytt. Scrob. Cord. et Cerat. Sabin.

22d.—Cough diminished; does not disturb him nearly so much at night; breath not altered; expectoration is purulent, not so large in quantity; is raised with less difficulty. Pr.

24th.—Expectoration still less; does not cough so much; breath nearly as before; appetite improved. Pr.

27th.—Breath rather better; is stronger; coughs less; cough does not occasion any pain; it chiefly occurs when he first awakes in the morning; bowels regular; appetite not quite so good. Pr.

31st.—Breath rather worse yesterday, but better again this day; wheezing has not abated; describes it as being close close to his throat; appetite and strength as last described; expectoration scarcely so purulent; a small tinge of blood in one part; cough, if at all different, is rather harsher; tongue clean, excepting at the back part, where it is a little covered; bowels rather confined; pulse quick and small. Pr.

Pil. Al. c. Myrrh j. alt. noct.

Empl. Lytt. Sterno (prope guttur) et Cerat. Sabin.

Feb. 3d.—Rather better; tongue clean; cough diminished and not so harsh; expectorates in the morning only; expectoration thick and yellow, not exceeding a quarter of a wine-glass full in 24 hours; bowels opened about twice; blister does not now discharge much; has drawn well. Pr.

5th.—Cough and breath better; expectoration diminished, thick, and yellow; by a pill taken the night before last, his bowels were opened four times yesterday; this has weakened him: blister nearly well. Pr.

7th.—During the last two days cough increased and appetite diminished; does not wheeze much; still feels (what he calls) some stoppage at the lower part of his throat; expectoration nearly as last report; tongue clean; bowels regular; pulse quick, but not hard or full; blister quite well. Pr.

Empl. Lytt. Stern. prope guttur, et Cer. Sabin.

14th.—On the whole materially better; cough much diminished, but scarcely to the same degree as before the 5th instant; appetite as last report; scarcely any stoppage in his throat; pulse quick and small; blister well about two days. Pr.

16th.—Coughed much yesterday and last night; very little this day; expectoration last night increased, but not this morning; throat not at all stopped; appetite nearly as it was. Pr.

17th.—Discharged.

Case V.—George Bell, hatter, aged 55, residing at No. 9, Meeting-house-walk, Snow's Fields; ill four months; admitted Jan. 30th.—Dyspnœa, cough, and expectoration, considerable; the mucus is expectorated with difficulty; coughs most severely when he first lies down at night, afterwards not so much; feels best in the morning; is much thinner than formerly; does not perspire; appetite very good; not thirsty; much flatulence; bowels regular; pulse moderate, occasionally intermittent. In summer is totally free from disorder of the chest, and from flatulence. Has been ill in winter during the last ten years. His complaints are aggravated when the weather is particularly severe, still more so when it is foggy; they are also increased by constipation of the bowels. Was hindered the last and the present winter, by the severity of his complaints, from working at his business. Em. Ol. Ant.

31st.

31st.—Has lost his hearing since his admission; was deaf every winter till the present; cough somewhat lessened; breath as it was; tongue a little brown; bowels regular; what he expectorates is clear mucus; it is not so large in quantity this morning as yesterday; has lost his appetite since he has been here; no irregularity in the pulse. Pr.

Feb. 3d.—Scarcely coughs at all; does not expectorate above one-fourth part of what he did; this comes up very easily; hearing much amended; breath considerably better; tongue clean; appetite increased; mouth feels sore since yesterday afternoon; pulse regular.

Aq. Menth. pip. ℥j. ter die.*

5th.—Still better; internal part of the lips excoriated, an very sore; bowels regular; appetite good; deafness almost gone; expectoration trifling. Pr.

7th.—Coughs scarcely at all; mouth better; bowels regular; very little expectoration; appetite good; still very weak, yet he thinks himself a little stronger. Pr.

14th.—No cough whatever; breath not affected, excepting in the evening, and then only in a trifling degree; mouth nearly well; bowels confined; some swimming in the head. Pr.

Pil. Al. c. Myrrh j. o. n.

16th.—Has not had the pills; giddiness in head; bowels confined. Pr.

Pil. Aloes c. Myrrh j. o. n.

19th.—Still better; the pills scarcely produce any effect. Pr.

P. Coloc. vice Pil. Aloe c̄. Myrrh.

20th.—Discharged by his own desire.

Case VI.—William Tonks, silversmith, aged 61, residing at No. 26, Bond-street, Borough Road, near the King's Bench; ill three months; admitted Feb. 17th.—Breath excessively short; coughs and spits very much; expectoration said to be thick tough phlegm, nearly a pint in 24 hours, raised with considerable difficulty; wheezes; has no pain any where, though he formerly had at the pit of the stomach; can scarcely walk; sometimes trembles violently; hoarse; appetite moderate; bowels regular; pulse moderate, weak. He coughs chiefly in the night, in consequence of which his rest is extremely bad; his breath is then sometimes so much affected, that it can scarcely be drawn. In the summer he is very well; was ill six weeks last winter; is now so bad as to be entirely incapable of working.

Empl. Lytt. Scrob. Cord. et Cer. Sabin.—Em. Ol. Ant.

* We insert this from the author's manuscript, though we suspect there must be an error. Ed.

19th.

19th.—Very little alteration; the expectoration is very frothy; no evacuation during the last two days; blister quite well; pulse small, and irregular in the extreme.

Empl. Lytt. Stern. et Cer. Sab.
Liq. Ant. Tart.
P Coloc. j. o. n.

21st.—Rather better; coughs so much in the night that he cannot rest; breath rather better; bowels regular; expectoration nearly the same in quantity and appearance. Pr.

23d.—Cough and expectoration as before; breath a little easier; bowels regular. Pr.

28th.—Better; cough not so bad; slept, he believes, six hours last night; has not had so good a night's rest during the last month; has rattling in his throat; tongue covered, but the covering loose and moist; expectoration scarcely so great; raised more easily; spirits better; blister discharges. Pr.

March 2d.—Still better; breath improved; stronger; much tickling in his throat; about five hours sleep last night; expectoration frothy. Pr.

5th.—On the 2d at night he became extremely ill during the whole night. Mr. Key, who visited him, told me that his pulse was very intermittent, and his breath excessively short. Mr. K. gave him two colocynth pills, as his bowels were confined: when they were opened he became better. Now is nearly as last report: complains much of the tickling in his throat; bowels open; pulse regular; blister discharges but little. Pr.

Empl. Lytt. prope guttur et Cer. Sabin.

7th.—The night before last he coughed and expectorated much; last night rested very well; at neither time were his bowels confined; blister has risen well; has not nearly so much wheezing; tongue moist, but still somewhat covered; does not spit so much; pulse regular, but rather quick. Pr.

9th.—Wheezing exactly as last report during the night; better in the day; tongue cleaner; bowels regular; six or seven hours sleep last night, though not without some interruption; spits less; pulse regular; blister discharges much, but being very small, I ordered

Empl. Lytt. Sterno et Cerat. Sabin. Pr.

12th.—Better; blister discharges; scarcely coughs except on lying down and on getting up; rattling still continues; expectoration further diminished; is still frothy; bowels regular; tongue nearly clear; appetite good; stronger. The weather became colder the day before yesterday. Just at the change his cough and dyspnœa were increased. Before coming hither he used to know perfectly well when the weather was about to change. Does not at this moment feel at

 all all worse, though the wind is north and the snow lying on the ground. Pr.

14th.—Was not so well yesterday; coughed much then and the night preceding; expectorated more than usual; tongue nearly clean; no pain in chest; pulse and bowels regular; blister discharges much; appetite continues good. Pr.

16th.—Coughed and expectorated much yesterday; two evacuations yesterday; the day before the bowels were confined; expectoration of the same kind, but diminished in quantity; blister discharges much. Pr.

19th.—Cough worse yesterday and this day; blister dry; wheezes a little; stronger; tongue scarcely at all covered; bowels regular. Pr.

Empl. Lytt. Stern. et Cer. Sabin.

21st.—Blister was put on last evening; slept better last night than since being here; expectorates still less; expectoration evidently mucus, though still somewhat frothy; appetite good; strength greater; appearance has gradually improved; says he is better in every respect. Pr.

Discharged.

Case VII.—Thomas Barnes, brewer, aged 45, residing at No. 89, Red-Cross-street, near Park-street, Southwark; ill four months; admitted Feb. 17th.—Cough very severe; breath extremely short; sometimes expectorates very much, always with great difficulty; gives him pain all over his chest when he coughs, particularly in the left side; feels this pain if he turns in his bed; cough and other symptoms aggravated at night; hence he is almost totally deprived of rest; during the night sweats much both when he sleeps and when he coughs; becomes thinner; extremely weak; tongue rather white; bowels regular; appetite moderate; thirsty; pulse quick and small. Illness commenced this winter with the cold weather; he feels worse when the weather is cold or foggy. He has not worked during six weeks; has had this complaint several winters, but is very well in the summer.

Em. Ol. Ant.
Empl. Lytt. Hyp. sin. et Cer. Sabin.

19th.—Has not had the remedies I prescribed; no better; bowels confined; expectoration appears to be thick mucus, of which he brings up about half a pint in twenty-four hours.

Pil. Coloc. j. o. n.
Liq. Ant. Tart.
Empl. Lytt. Hypoch. sin. et Cerat. Sabin.

21st.—Not so much pain in his side; cough as it was; pain pain in his loins; blister discharges much; has not had the pills.

Rep. Liq. Ant. Tart.

Pil. Al. c. Myrrh j. o. n.

Empl. Pic. Comp. Lumb.

23d.—Cough, breath, and expectoration, rather better; bowels open once daily; not so much pain in the loins; no pain in his side; complains much of weakness; tongue more moist, but rather white; blister discharges.

28th.—Blister discharges; he is better; cough diminished; still wakes him occasionally at night; expectoration raised with difficulty, less in quantity, and nearly the appearance of healthy mucus; tongue clean; appetite moderate; rather stronger; bowels regular; the night before last coughed more than usual. Pr.

March 2d.—Better; still some pain in his chest when he coughs; cough affects him chiefly in the morning, when the little mucus which he now expectorates is brought up; slept about four hours last night; bowels regular.

Empl. Lytt Sterno et Cerat. Sabin. Pr.

5th.—Very poorly yesterday and this day; cough better; breath not worse; expectoration the same, but feels very low and weak, and his appetite is not quite so good; tongue clean; bowels regular; blister discharges. Pr.

7th.—Better; not so low; expectoration raised with difficulty; not so much in quantity; slightly tinged with blood; before coming hither raised much blood; tongue clean; bowels regular; appetite moderate; pulse rather quick; blister discharges. Pr.

9th.—Better; slept about five hours last night without waking; expectoration diminished, but in it there are distinct lumps of blood or of mucus deeply tinged with blood; coughs but little; scarcely any pain when he coughs; appetite improved; blister discharges. Pr.

12th.—Blister discharges; still spits blood; appetite better; strength nearly as it was; bowels regular. Pr.

14th.—Better; no blood last night, nor scarcely any expectoration. Pr.

16th.—Stronger; expectoration trifling; a very small streak of blood in one place only; sleeps very well; bowels regular. Pr.

19th.—Stronger; coughs scarcely at all; still a little blood; slept badly last night, yet had no cough; bowels regular; appetite good. Pr.

21st.—Still better; walked out yesterday; coughed this morning morning no more than usual; no blood expectorated; stronger; blister well.

Empl. Picis Comp. Sterno.

Discharged.

CASE VIII.—Patrick Goad, laborer, aged 34; ill five months; admitted February 20th.—Coughs severely; breath very short; if he uses any exertion can scarcely breathe; some pain in his chest when he coughs; expectoration about a quarter of a pint in twenty-four hours, is brought up with great difficulty, and after severe efforts; rather hoarse, but not so much so as he has been; great pain across his loins; pain in his head; no stool during the last nine days; extremely weak; does not sweat; wastes away; pulse small; tongue rather white; appetite not bad; rather thirsty. Complaints are aggravated by cold or foggy weather, or by lying on his right side. His disorder first began three years ago, in consequence of a severe cold: since then he has been ill at different times, chiefly in the winter: during the last he was not nearly so bad as he now is, and during the summer was very well. In September his complaints recommenced, and gradually increased until Christmas, when they were so severe as to render him quite incapable of working. For some weeks past he has in vain tried to gain admission into the London Hospital. During this time he has been under medical treatment, but has derived no benefit from it.

Liq. Ant. tart.
Pil. Coloc. j. o. m.
Empl. Lytt. Scrob. Cord. et Cer. Sabin.

21st.—Rested last night rather better than usual; blister has risen; cough and breath the same; the expectoration is mucus. Pr.

23d.—Better; slept pretty well last night; cough, breath, and expectoration, better; expectoration raised more easily; tongue moist, but rather white; very weak in the loins; blister discharges. Pr.

28th.—Much better; cough and expectoration diminished; expectoration nearly like common mucus; occasionally flying pains in his right side; complains of his loins; stronger; bowels regular; blister discharges. Pr.

Empl. Pic. Comp. Lumb.

March 2d.—Loins better; coughs little; expectoration trifling, and nearly natural; occasional pain in the right side; sometimes it continues for two days, and sometimes it does not attack him, perhaps, during four or five; sleeps five or six hours without waking; bowels regular; blister not yet well. Pr.

 5th.

5th.—Better in every respect; coughs and spits scarcely at all, but chiefly in the morning; stronger; blister quite well. Pr.

7th.—Still better; bowels regular; tongue a little covered and yellow. Pr.

9th.—Further improved; no expectoration. Pr.

12th.—Stronger; no tightness at his chest; bowels regular. Pr.

16th.—Stronger. Pr.

19th.—Very well. Nihil.

21st.—Very well.

Empl. Picis Comp. Sterno.

Discharged.

Case IX.—William Quin, sailor, aged 54; ill three months; admitted January 20th.—Coughs very severely, particularly at night; breath extremely short; worse than usual when he coughs; pain in his chest during the exertion of coughing, but not now so severe as it has been; expectorates considerably, about half a pint in twenty-four hours; expectoration raised with difficulty; no blood is spit up. These complaints are aggravated when the weather is foggy, when he lies down and when he rises, or when he quits a warm room for a cold situation. His night's rest is much disturbed, sometimes so much that he can scarcely sleep at all; has pain in all his limbs, chiefly in the right arm and leg; the left arm and leg were as bad, but now are better; no head-ach unless when he coughs severely; becomes very thin; does not perspire; very weak; appetite good; not thirsty; bowels confined; tongue clean; pulse small and quick. Was perfectly free from complaint till the beginning of the winter, when he arrived in this country from the West Indies, after a very boisterous passage, during which he was almost constantly wet, and had not sufficient change of cloathing. Being wholly unable to work at Christmas, he attempted to gain admission into the London Hospital, but without success. From that period till the time when I admitted him into the room in Horselydown, he received medicines from the hospital, and was somewhat relieved whilst using them.

Pil. Coloc. j. o. n.

Liq. Ant. tart.

Empl. Lytt. Scrob. Cord. et Cer. Sabin.

21st.—Rested better last night than he has done for some time; cough and breath relieved in a trifling degree; the expectoration in general has the appearance of mucus; some part might perhaps be denominated puriform mucus. Pr.

Lin. Ammon. b.d. brachio.

23d.

23d.—Cough, breath, and expectoration, better; pains in limbs somewhat relieved; rests very well at night; coughs chiefly in the morning; bowels regular. Pr.

28th.—Better in every respect; expectoration diminished, appears like mucus; raised still with difficulty; sleeps soundly at night; bowels regular; tongue a little covered, but moist; pulse, though small, regular and good; blister discharges. Pr.

March 2d.—Blister discharges a little; right arm somewhat better; coughed much last night, but did not expectorate at all; says he has a stoppage at his chest near the throat. Pr.

5th.—Blister quite well; coughs scarcely once during the day; stoppage in the chest last night; on drawing his breath deeply, experiences some pain in the left sides of the Scrob. Cord. but not nearly so much as he used to feel; arm better. Pr.

Empl. Lytt. Scrob. Cord. et Cer. Sabin.

7th.—Blister discharges well; better in every respect. Pr.

9th.—Cough rather worse last night; cannot expectorate; feels some oppression at his chest when he draws his breath. Pr.

12th.—Better; coughs in the morning, and then expectorates, but at no other time; arm better; bowels regular; the blistered surface is at the upper part of the chest, and he feels the tightness chiefly at the pit of the stomach. Pr.

Empl. Lytt. Scrob. Cord. et Cerat. Sabin.

14th.—Still a little pain in chest when he coughs; expectoration also still confined, with some tightness at the sternum; expectoration trifling; cough as last report; arm better. Pr.

16th.—Coughed much this morning; tightness at sternum continues; nearly as before the last blister; arm better; when he was admitted could not raise it to his head, now can move it with ease in almost every direction. Pr.

19th.—Threw up some blood last night; likewise coughed much; blister quite well. Pr.

Empl. Lytt. Scrob. Cord. et Cerat. Sabin.

21st.—Blister discharges; no blood whatever; arm still better. Pr. Discharged.

The cases just described were all which I received into this room. They were taken almost at random as I was able to procure them, the proposal of remaining there for some weeks having been made to many individuals who refused to accede to it. The publication of all the circumstances in detail I considered as the fairest mode of enabling medical medical men to form a judgment of the benefits to be derived from adopting this plan. Were there no other instances on record besides the above, it appears to me that I might fairly infer from them that the remedy is of considerable importance and utility in the treatment of pulmonic diseases. Yet it must be granted that the different individuals who were the subjects of this treatment were benefited in very different degrees. All of them, excepting Osborn (Case 1st) and Hughes (3), were so severely ill as to be totally incapable of working, and even these worked with considerable difficulty. When discharged they were all still weak, but very much stronger than when admitted; yet they appeared to me to make less improvement in regard to strength, than they did in any other respect. Bell (5) and Good (8), when discharged, had no symptoms whatever of pulmonic disease; Hughes (3) and Quin (9) only a slight shade remaining; Barnes (7), Osborn (1), and Ellis (2), were not so much relieved; and Tonks (6) and Corney (4) least of all. Yet even these were considerably improved: Tonks was much stronger than when admitted, and could sleep throughout a whole night without being waked once by his cough; Corney neither coughed nor expectorated nearly so much as he had done, and his strength was much greater than on his admission. Had some of the patients continued longer in the apartment, I have no doubt that the benefit received would have been more strongly marked. Their longer continuance, however, was inconsistent with the plan I at first proposed, which was, to keep each individual four weeks in that situation. Excepting in two instances, this intention was carried into effect. Bell (5) was so much recovered at the end of three weeks that he desired to be discharged; and Ellis (2), by the severity of his disease, his weakness, and his poverty, I was induced to continue ten days longer than the appointed time.

It will, perhaps, be observed, that the pure experiment of the efficacy of an individual remedy was not here tried, as other means of very considerable power were employed at the same time. That other and powerful means were used, is true; and, without doubt, the amendment of the patients must, in a considerable degree, be attributed to these efficacious auxiliaries; yet I think I may say that in three of the instances a pure experiment was fairly and fully tried—I refer to Bell, Corney, and Hughes. All the medicine which Bell (5) took, was for three or four days some oily emulsion with antimony, and subsequently a tea-spoonful of peppermint water three times a-day. His bowels were likewise kept regular. No one can possibly conceive that these these articles were of material consequence to his relief. Corney (4) and Hughes (3) had been using remedies of the same description several months before their admission, as they did subsequently; yet they had been relieved only to a certain degree, and then became nearly stationary. Hughes might be considered as scarcely having any symptoms of pulmonic disease when he was discharged, his progress towards recovery having been much greater in the four weeks since his admission, than it had been in as many months previously. Corney likewise was materially better than when admitted, though perhaps less relieved than any of his companions. Besides these three, Goad (8) and Quin (9) had previously been using medicines under my direction, though not with the same degree of constancy and attention as the two last. The former, i. e. Goad, derived no benefit from them, and the latter very little; but, subsequent to their admission, the relief of each, particularly of Goad, was extremely great. Comparing these nine individuals with patients of the same description under my care at the same time in the London Hospital and in other places, the difference between them was very striking. The latter were lingering on, while the former were, in general, rapidly amending. All were using the same remedies excepting the regulated temperature. The difference, therefore, could only be attributed to this one remedy being employed in some instances and not in others.

Two persons, to whom I had recommended a residence in this room, but who did not think proper to comply with my recommendation, are since dead; and I think it very probable that some of those who were admitted, would not now have been alive had they not received the assistance which was thus offered them.

It is not to be expected that artificial heat will produce more benefit to patients affected with pulmonic disease, than the recurrence of summer warmth will effect in their behalf; but, as summer gales cannot, during the winter months, be commanded, the best substitute that can be procured must be resorted to in their stead, and, if employed, will be found a powerful remedy.

